# Intratumoral microbial abundance and load influence the immune microenvironment of oral squamous cell carcinoma

**DOI:** 10.3389/fonc.2025.1616928

**Published:** 2025-09-22

**Authors:** Jiajun Fang, Mengna Wu, Hongyu Shen, Weijie Liu, Tonghan Zhang

**Affiliations:** ^1^ Department of Oral and Maxillofacial Surgery, Hospital of Stomatology, Zhongshan, China; ^2^ School of Stomatology, Jinan University, Guangzhou, China

**Keywords:** oral squamous cell carcinoma, intratumoral microbiome, tumor infiltrating lymphocytes, immune checkpoints, immunotherapy

## Abstract

**Objective:**

This study aimed to explore the characteristics intratumoral microbiome in oral squamous cell carcinoma, and elucidate the interplay between intratumoral microbial profiles (relative abundance/absolute load) and tumor-infiltrating lymphocytes markers (CD4^+^/CD8^+^/FOXP3) and PD-L1 in oral squamous cell carcinoma

**Methods:**

We analyzed 45 OSCC tissue samples alongside paired paracancerous (n=10) and normal oral microbiota controls (n=8). Microbial composition was characterized by 16S rRNA sequencing (V3-V4 regions), with bacterial load quantified via qPCR targeting the V4–1 region. Tumor-infiltrating lymphocytes markers were assessed by immunohistochemistry.

**Results:**

*Spirochaetota* was concentrated in the CD4^+^ as well as CD8^+^ low infiltration groups, *Proteobacteria* in the CD8^+^ high infiltration group and *Actinobacteriota* in the FOXP3 low infiltration group. Tumor microbial load was negatively correlated with CD4^+^, CD8^+^, and FOXP3, but of these, only the correlation coefficient of -0.309 for CD4^+^ was statistically significant. However, no significant correlation was observed in the analysis of PD-L1 expression with the relative abundance of intratumoral microbiome, α-diversity, and intratumoral microbial load values

**Conclusion:**

Changes in the abundance of specific intratumoral microbiome affect the infiltration of TILs markers, and there is a negative relationship between intratumoral microbial load and T-cell infiltration, suggesting that intratumoral microbiome contribute to the processes of the tumor immunosuppressive microenvironment.

## 1 Introduction

Oral squamous cell carcinoma (OSCC) is a malignant tumor arising from the mucosal epithelium with squamous metaplasia, affecting anatomical sites including the buccal mucosa, gingival mucosa, retromolar trigone, floor of the mouth, tongue (anterior two-thirds), hard palate, and lips (1). OSCC constitutes 90% of all oral malignancies. In 2020, global statistics reported 377,713 new cases and 177,757 deaths (2), with incidence rates steadily increasing over the past 30 years (3). The prognosis remains dismal, with a 5-year survival rate of only 50% (4). Traditional risk factors include tobacco use, alcohol consumption, betel quid chewing, and genetic susceptibility (1). However, growing evidence highlights the tumor microbiome as a critical player in OSCC development. Hallmarks of Cancer updated in 2022 now recognizes polymorphic microbial communities as a defining feature of tumors, capable of potentiating oncogenic hallmark traits (5).

Notably, few microorganisms directly induce malignant transformation. Instead, many collaborate with the host immune system to facilitate tumor progression (6). The oral microbiota may promote OSCC initiation and development both directly and indirectly by exacerbating chronic inflammation and oxidative stress (7).In a landmark 2020 study, Nejman et al. confirmed the existence of intratumoral microbiome, demonstrating that every tumor type harbors unique microbial communities primarily localized within cancer cells and immune cells. Subsequent research has increasingly established correlations between intratumoral microbial dysbiosis and oncogenesis (8). intratumoral microbiome constitute diverse ecological communities within neoplastic tissues, encompassing bacteria, fungi, mycoplasma, viruses, and parasites (9). Although bacterial cells account for only ~3% of total tumor cellularity (10), these low-biomass populations critically influence the tumor microenvironment (TME). They remodel the TME through multilayered mechanisms (11), with emerging evidence showing that intratumoral microbiome and their metabolites can modulate the composition and functionality of tumor-infiltrating lymphocytes (TILs). For instance, *Fusobacterium nucleatum* in breast tumors suppresses TIL recruitment while accelerating metastatic progression (12). Of particular clinical relevance, PD-L1—a key immune checkpoint protein mediating tumor immune evasion—remains the only validated biomarker for immunotherapy selection in recurrent/metastatic HNSCC (13).

The purpose of this study was to describe the effects of intratumoral microbiome on TILs and PD-L1 in the tumor microenvironment of OSCC patients. By comparing the abundance and load of intratumoral microbiome, the infiltration levels of TILs markers (CD4+T cells, CD8+T cells, and FOXP3), and the expression of PD-L1 in different OSCC tissues, we explored the potential impact of intratumoral microbiome on the immune microenvironment, and explored its potential value as a diagnostic biomarker for OSCC.

## 2 Materials and methods

### 2.1 Patient cohort and sample collection

Samples were collected from the Department of Oral and Maxillofacial Surgery of Hospital of Stomatology, Zhongshan City, China, between January 2022 and December 2024 from patients with the clinical diagnosis of OSCC by pathology. A total of 46 samples were collected for the study, all samples collection processes were strictly carried out in accordance with the specifications, and the informed consent of the patients was obtained and approved by the hospital ethics committee. Among the samples, 10 fresh tumor and adjacent tissue samples were obtained in the operating room, and the other 36 cases of OSCC tissue were collected from samples preserved in the Pathology Department. Eight normal oral microbial control groups were taken for comparison (distal gingival flap obtained from outpatients with impacted third molars extracted without localized inflammatory state).

### 2.2 Microbiome testing

The total DNA of 63 samples (45 OSCC samples, 10 paraneoplastic control samples and 8 normal tissue control samples) was further processed for amplicon-based sequencing of the highly variable region of the 16S rRNA gene V3-V4. The raw image data files obtained from sequencing were converted into Raw Reads by Base Calling analysis, and the raw sequencing data were spliced for quality control to obtain Operational Taxonomic Units (OTUs), which were compared with the Silva16srRNA database (v138), and then species classification, abundance analysis, α diversity analysis and β diversity analysis were performed.

The intratumoral microbial load was estimated by quantitative polymerase chain reaction (qPCR) to obtain bacterial DNA load. Equal amounts of DNA were used to perform qPCR with primers targeting V4-1 (515F: GTGCCAGCMGCCGCGGTAA, 806R: GGACTACHVGGGTWTCTAAT). The cutoff values for high and low counts were defined as the median of the patient group.

### 2.3 Immunohistochemistry

After the collected samples were sliced continuously, the corresponding tumor tissue pathological sections of each case were obtained, and immunohistochemical staining was performed using the EnVision two-step method. The primary antibodies included CD4+ (abcam, EPR6855), CD8+ (abcam, RM1129), FOXP3 (abcam, EPR22102-37) and PD-L1 (abcam, EPR19759), and the secondary antibodies were purchased from Beijing Zhongshan Jinqiao Company (PV 8000 kit). The experimental steps were strictly carried out according to the instructions of the kit. The interpretation of the sections was completed independently by 2 physicians, and the values were averaged. The readers were blinded to the clinical pathological data of the patients. PD-L1 was positive when yellow or brown particles appeared in the cytoplasm and (or) cell membrane. The CPS score was calculated. CPS ≥ 1 was classified as a high expression group, and CPS < 1 was classified as a low expression group. CD4+, CD8+ and FOXP3 cell membrane and (or) cytoplasmic staining of any intensity and any proportion was positive, and no staining was negative. Three fields of view were selected at high magnification (400 times), and the positive cells in the field of view were counted three times and the average was taken. The average of the three fields of view was taken as the final expression value. Expression values less than the median were classified as low expression group, and those greater than the median were classified as high expression group.

### 2.4 Statistical analysis

All the statistical data were collected and analyzed using SPSS 26.0 software package. Descriptive statistics of clinical and microbiological data were expressed as mean ± standard deviation (Mean ± SD). For normally distributed data, the independent sample t test was used to compare the differences between the two groups. Microbiological related data such as relative abundance of bacteria, absolute bacterial load, α diversity index (Chao1, Shannon and Simpson index) and β diversity were all non-normally distributed data. Non-parametric statistical methods, Kruskal-Wallis (KW) rank sum test, Wilcoxon rank sum test, and spearman correlation analysis were used. β diversity analysis was performed using principal coordinate analysis (PCOA) based on Bray-Curtis distance to evaluate and visualize the differences in microbial community structure between samples. The difference analysis of microbiome between groups was performed using LEfSe analysis. Correlation analysis was performed using Pearson chi-square test or Fisher’s exact probability method. All statistical calculations were performed using two-sided tests, with a test level of α = 0.05 and *P* < 0.05 for statistical significance.

## 3 Results

### 3.1 Participant characteristics

This study included 45 histopathologically confirmed OSCC patients (35 males [77.78%] and 10 females [22.22%]), with a mean age of 56.78 ± 11.72 years (range: 29–81 years). The primary tumor sites were distributed as follows: tongue (30 cases, 66.67%), buccal mucosa (6 cases, 13.33%), floor of mouth (5 cases, 11.11%), palate (3 cases, 6.67%), and gingiva (1 case, 2.22%). Pathological examination revealed well-differentiated (19 cases, 42.22%), moderately-differentiated (23 cases, 51.11%), and poorly-differentiated (3 cases, 6.67%) tumors. According to TNM staging, patients were classified as T1 (11 cases, 24.44%), T2 (16 cases, 35.56%), T3 (15 cases, 33.33%), and T4 (3 cases, 6.67%), with lymph node metastasis present in N1 (10 cases, 22.22%) and N2 (5 cases, 11.11%) patients, while no distant metastases were observed. Lifestyle factors included smoking (18 cases, 40.00%) and alcohol consumption (10 cases, 22.22%).

### 3.2 Composition and diversity of intratumoral microbiome

**Figure 1:**
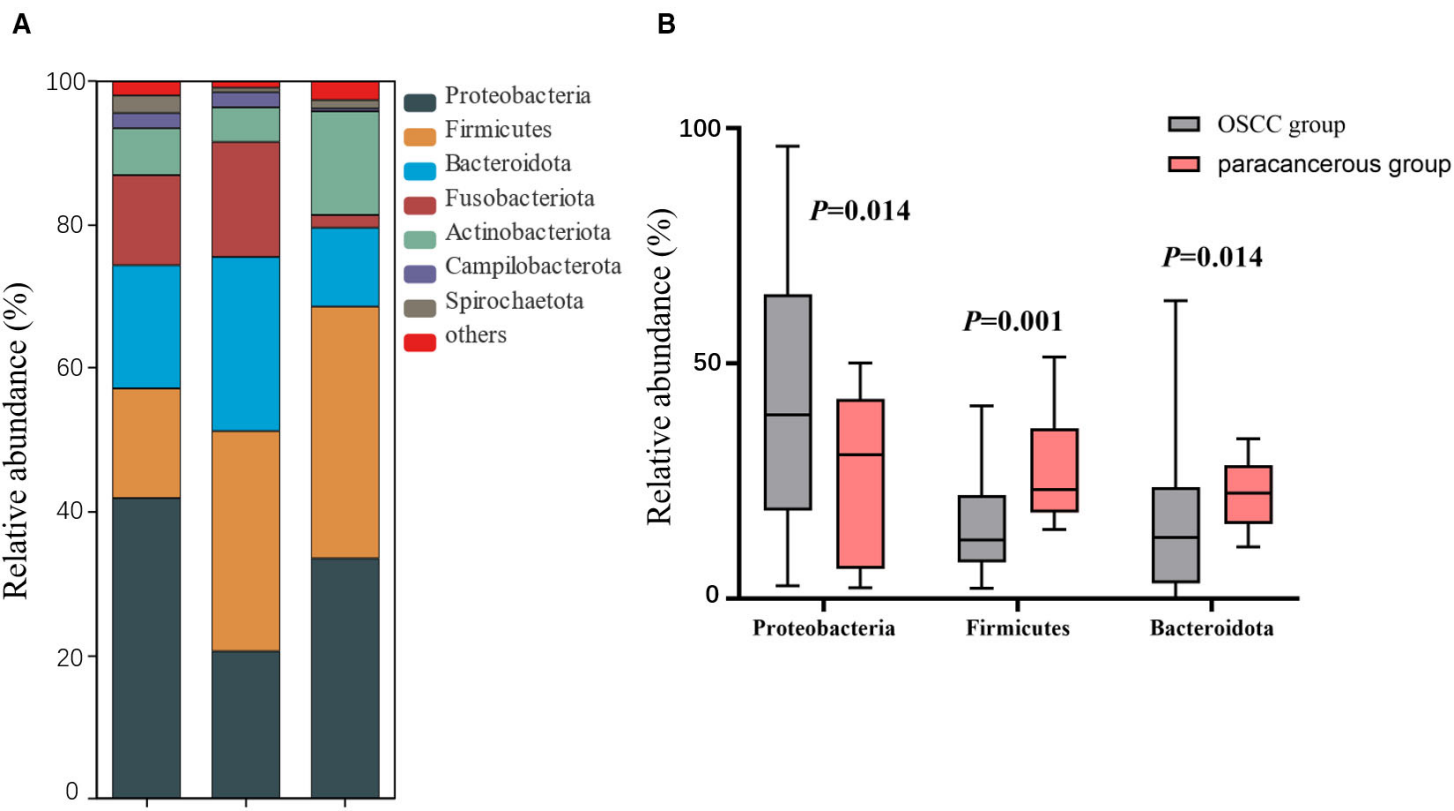
**(A)** Composition of dominant microbial colonies at the phylum level; **(B)** Difference between OSCC and paracancerous group at phylum level.

**Figure 2:**
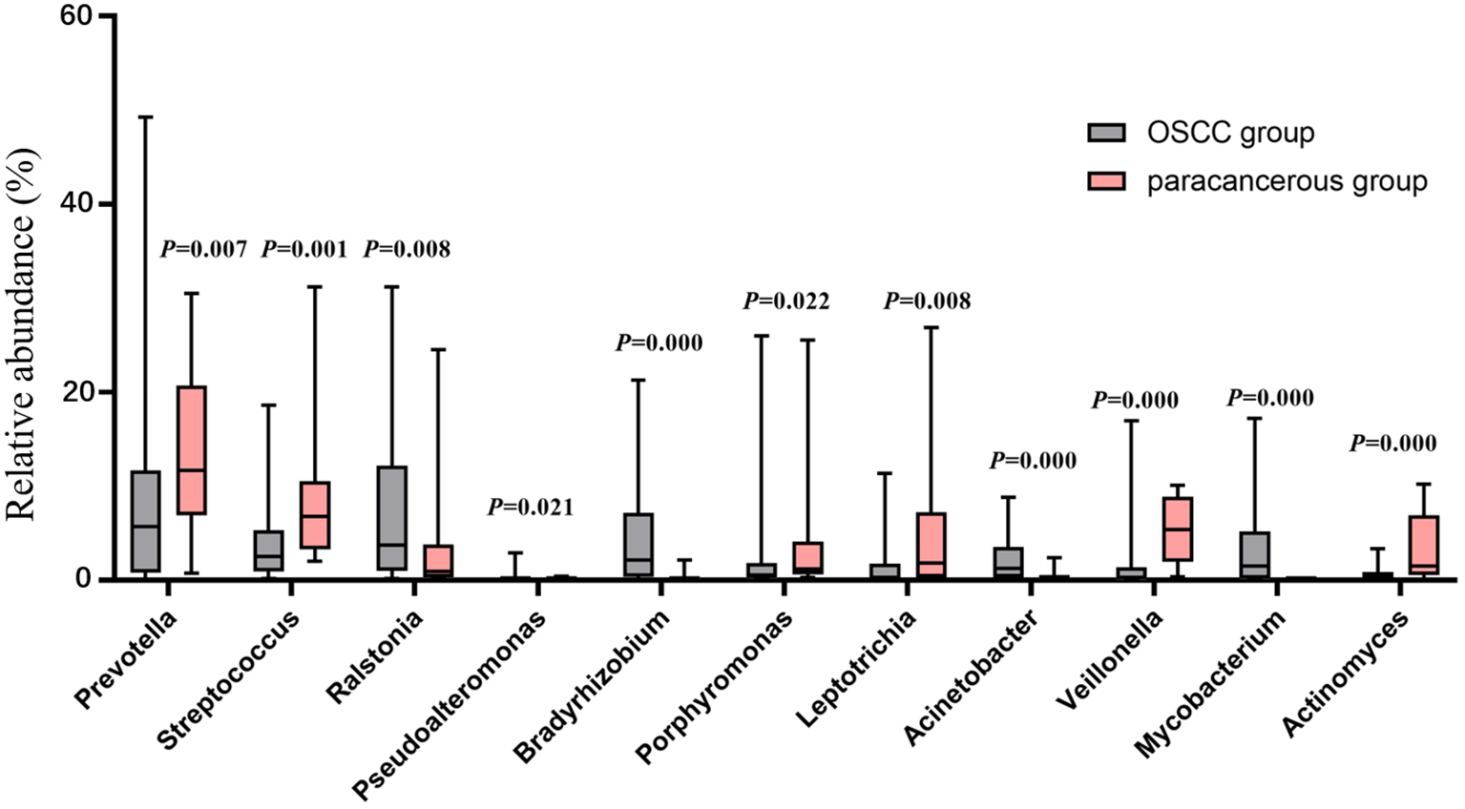
Difference between OSCC and paracancerous group at genus level.

At the phylum level, the dominant intratumoral microbiome in OSCC (abundance > 1%) were mainly *Proteobacteria*, *Firmicutes*, *Bacteroidota*, *Fusobacteriota*, *Actinobacteriota*, *Campilobacterota*, and *Spirochaetota* **Figure 1A**). The Wilcoxon rank sum test was used to screen out the differential phyla between OSCC and paracancerous group, and it was found that the relative abundance of *Proteobacteria*, *Firmicutes*, and *Bacteroidota* was significantly different **Figure 1B**). At the genus level, the Wilcoxon rank sum test was used to screen the differential bacterial genera between OSCC and paracancerous group, and it was found that the relative abundance of *Prevotella*, *Streptococcus*, *Ralstonia*, *Pseudoalteromonas*, *Bradyrhizobium*, *Porphyromonas*, *Leptotrichia*, *Acinetobacter*, *Veillonella*, *Mycobacterium*, and *Actinomyces* was significantly different (**Figure 2**).

**Figure 3:**
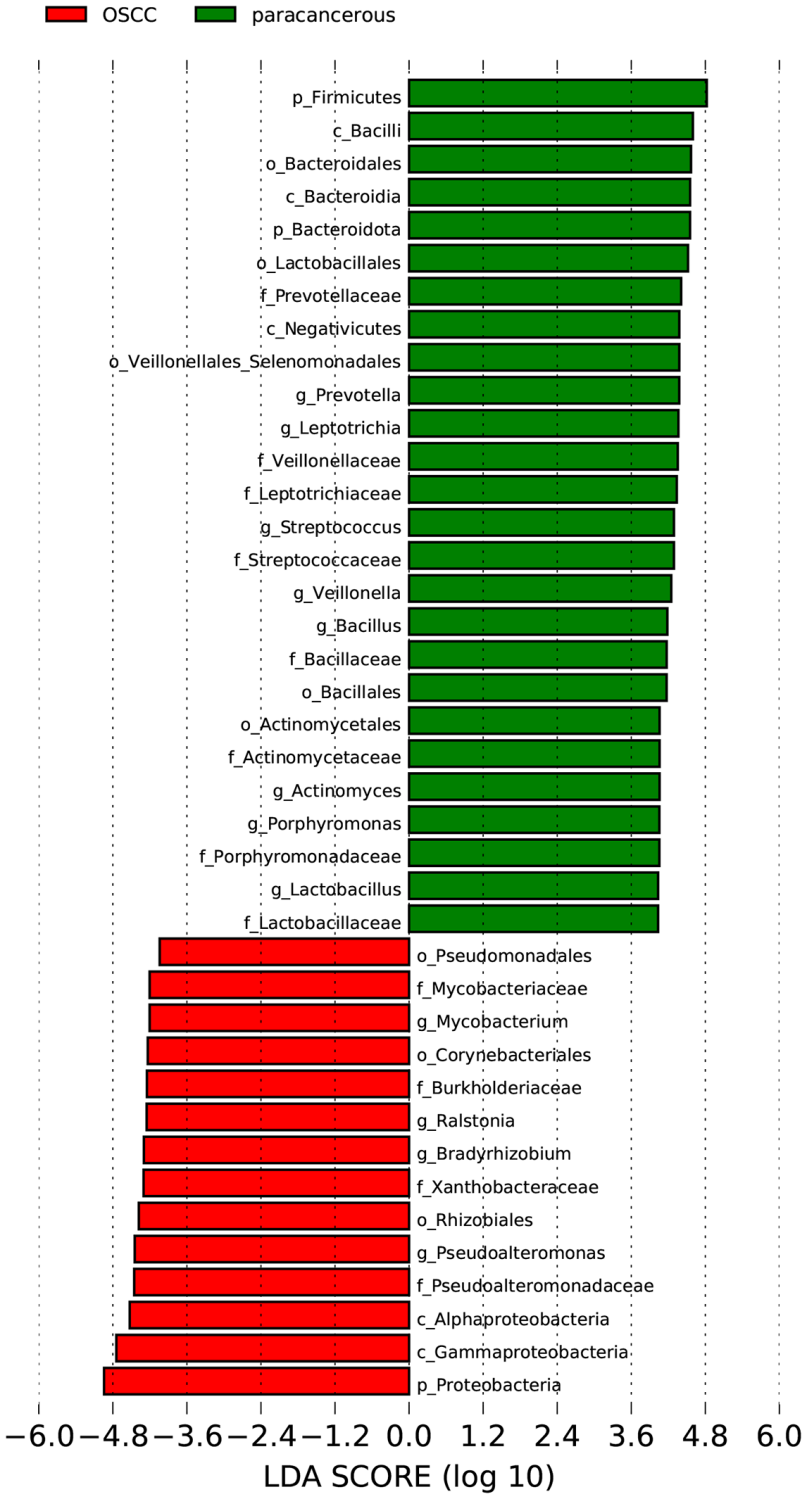
Differential abundances of core bacterial species among OSCC and paracancerous group.

**Figure 4:**
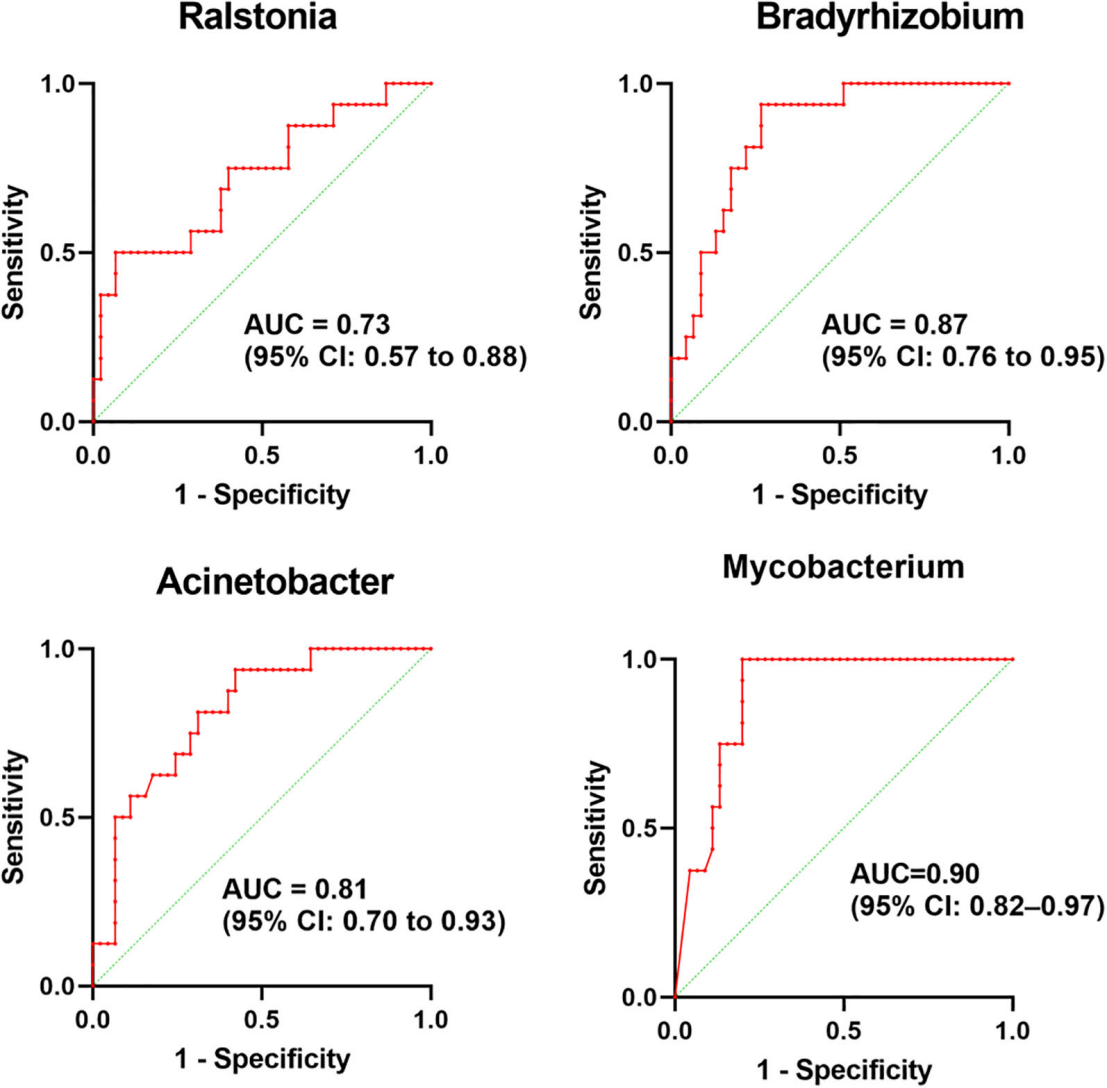
ROC curves evaluating the predictive potential of four bacterial genera as OSCC diagnostic biomarkers in the microbiome.

The LEfSe was used to analyze the bacterial communities with statistical differences between OSCC and paracancerous group, and to identify OSCC-related bacterial community markers. The results showed that in OSCC, the relative abundances of one phylum (*Proteobacteria*), two classes (*Gammaproteobacteria* and *Alphaproteobacteria*), three orders (*Rhizobiales*, *Corynebacteriales* and *Pseudomonadales*), four families (*Mycobacteriaceae*, *Burkholderiaceae*, *Xanthobacteraceae* and *Pseudoalteromonadaceae*) and four genera (*Mycobacterium*, *Ralstonia*, *Bradyrhizobium* and *Pseudoalteromonas*) were clustered. In the adjacent tissues, the relative abundances of two phyla (*Firmicutes* and *Bacteroidota*), three classes (*Bacilli*, *Bacteroidia* and *Negativicutes*), five orders (*Bacteroidales*, *Lactobacillales*, *Veillonellales_Selenomonadales*, *Bacillales* and *Actinomycetales*), eight families (*Prevotellaceae*, *Veillonellaceae*, *Leptotrichiaceae*, *Streptococcaceae*, *Bacillaceae*, *Actinomycetaceae*, *Porphyromonadaceae* and *Lactobacillaceae*) and eight genera (*Prevotella*, *Leptotrichia*, *Streptococcus*, *Veillonella*, *Bacillus*, *Actinomyces*, *Porphyromonas* and *Lactobacillus*) were clustered (**Figure 3**). Four of the most abundant and diverse genera, *Ralstonia*, *Bradyrhizobium*, *Acinetobacter*, and *Mycobacterium*, were selected for ROC analysis to evaluate the predictive potential of the microbiome as a diagnostic marker for OSCC, the results showed that *Ralstonia* had low accuracy, while *Bradyrhizobium*, *Acinetobacter* and *Mycobacterium* all had some accuracy (**Figure 4**).

**Figure 5:**
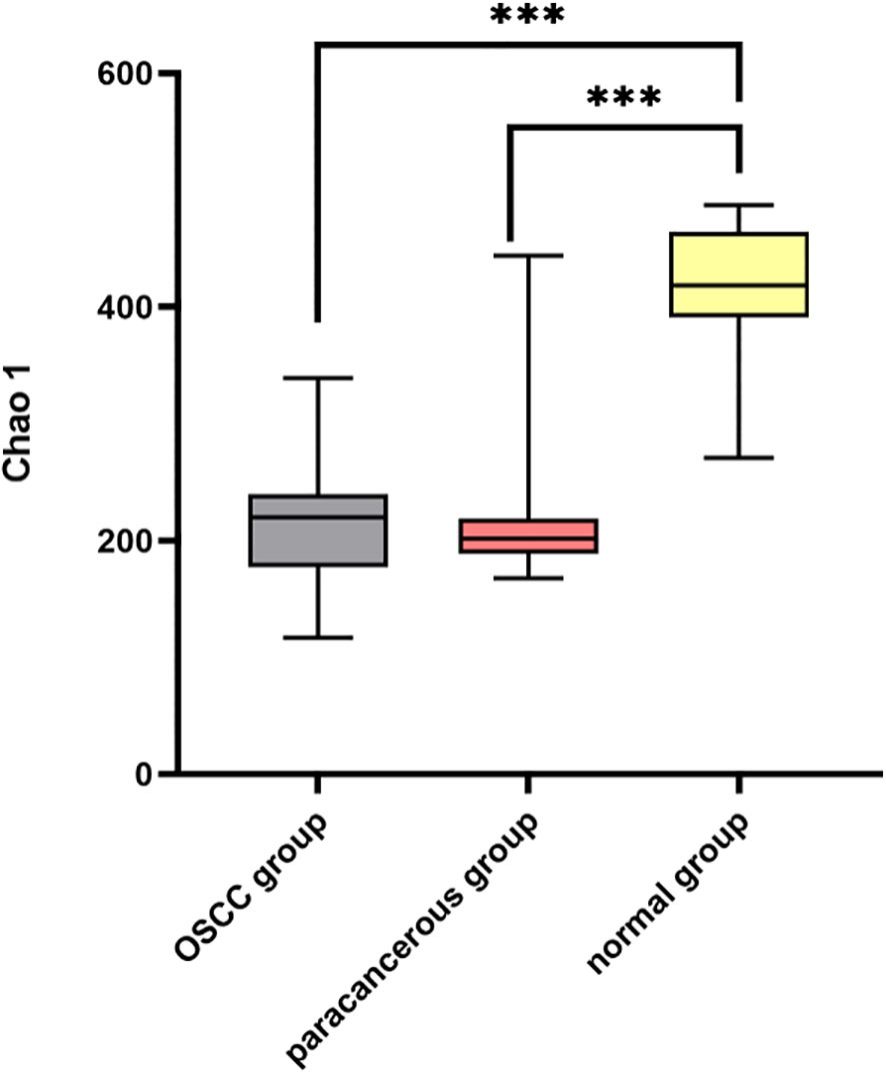
Comparison of Chao1 index of α diversity among three groups of samples.

**Figure 6:**
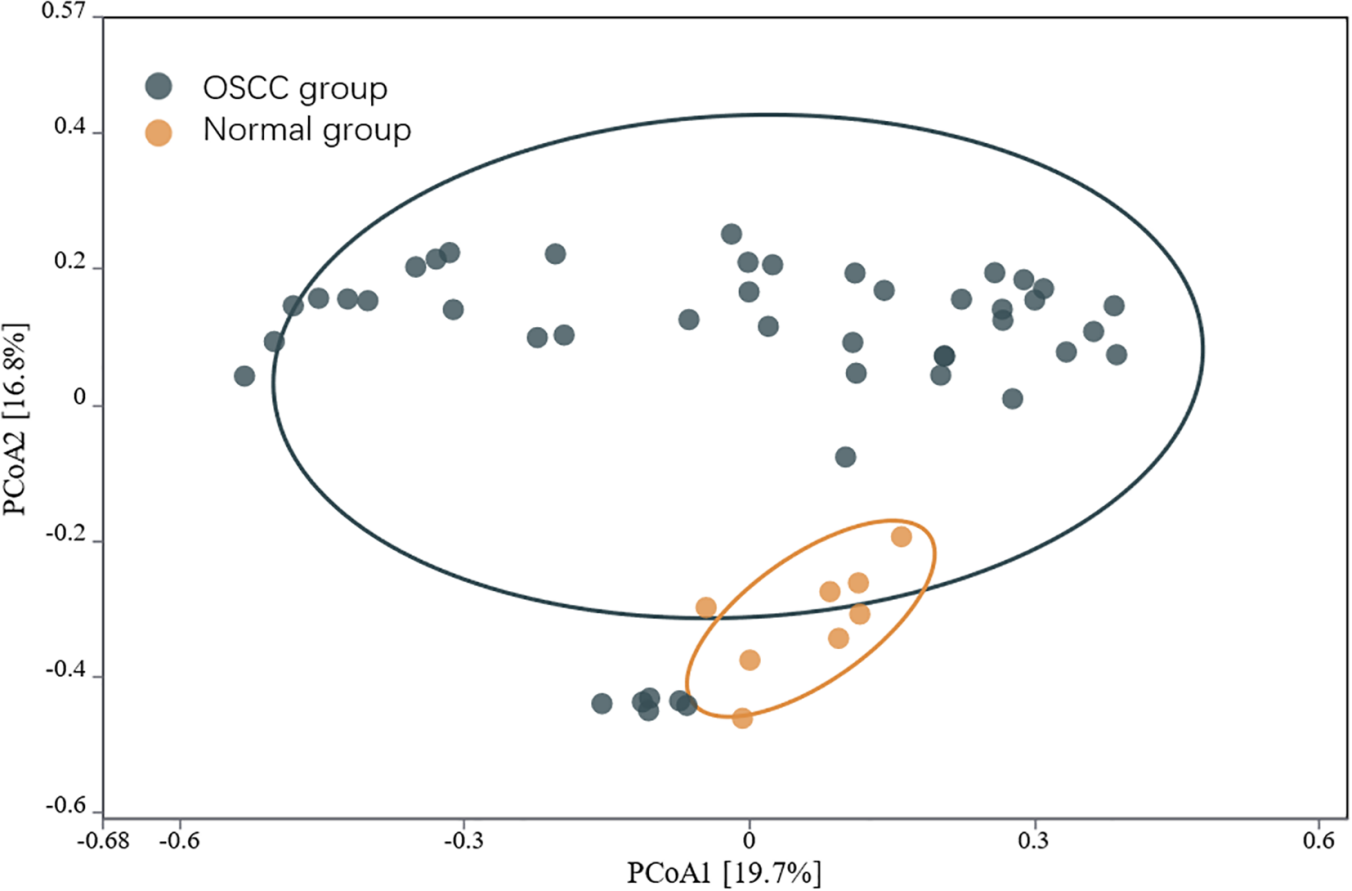
PCOA analysis of the microbiota of OSCC and normal control group based on the bray_curtis distance algorithm.

The α diversity of the intratumoral microbiome in the OSCC group, the paracancerous control group, and the normal oral microbial control group was compared by calculating the Chao1, Shannon, and Simpson indices. The results showed that Chao1, Shannon, and Simpson in the OSCC group were significantly reduced compared with the normal oral microbial control group (*P* < 0.05), while the difference in α diversity between the OSCC group and the paracancerous control group was not statistically significant (*P* > 0.05) (**Figure 5**). To further compare the differences between samples, the β diversity of the OSCC group and the normal oral microbial control group was analyzed. We used PCOA based on the bray_curtis distance algorithm to compare the differences in the OSCC group, paracancerous control group, and the normal oral microbial control group. It was also statistically analyzed by analysis of similarity (ANOSIM, Analysis of similarity) and found that there was a significant difference between the samples in the OSCC group compared to the normal oral microbiology control group (*P* < 0.05) (**Figure 6**).

### 3.3 Relationship between intratumoral microbiome and clinical characteristics in OSCC

We analyzed the composition of microbiome in groups according to different clinical characteristics. The results of gender grouping showed no statistically significant difference in bacterial abundance between the two groups. Tumor location (tongue, buccal, floor of mouth, palate, gingiva, and oropharynx) grouping showed no statistically significant difference in bacterial abundance between sites. The results of grouping by degree of differentiation showed a significant difference in the abundance of *Spirochaetota* (*P* < 0.05). The results of grouping by T-stage showed a significant difference in the abundance of *Actinobacteriota* (*P* < 0.05). The results of lymph node metastasis grouping showed a significant difference in the abundance of *Actinobacteriota* (*P* < 0.05). In addition, results on whether OSCC risk factors smoking and alcohol consumption affect microbial diversity and composition showed that the relative abundance of *Actinobacteriota* was significantly lower in patients with a history of smoking as well as with a history of alcohol consumption (*P* < 0.05).

### 3.4 Functional prediction of OSCC intratumoral microbiome

In this study, the Kyoto Encyclopedia of Genes and Genomes (KEGG) signaling pathways that may be involved in OSCC lesions were predicted through the analysis results of PICRUSt. KEGG enrichment analysis showed that the pathways significantly enriched in OSCC-related oral tissue microbial flora included Biosynthesis of ansamycins, Valine, leucine and isoleucine biosynthesis, D-Glutamine and D-glutamate metabolism, Biosynthesis of vancomycin group antibiotics, Fatty acid biosynthesis, C5-Branched dibasic acid metabolism, Peptidoglycan biosynthesis, Pantothenate and CoA biosynthesis, One carbon pool by folate, and D-Alanine metabolism.

### 3.5 Relationship between intratumoral microbiome and TILs in OSCC

To evaluate the relationship between intratumoral microbial abundance and diversity with infiltration of TILs markers and PD-L1 expression, we compared the percentage abundance of intratumoral microbiome at the phylum level with different infiltration of TILs markers (CD4, CD8, and FOXP3) and with different expression of PD-L1 (CPS <1 and CPS ≥1), and by Chao1, Shannon and Simpson indices to compare the α diversity of intratumoral microbiota. The results showed that intratumoral microbial α diversity was not significantly associated with high or low CD4^+^, CD8^+^ and FOXP3 infiltration and PD-L1 expression (*P* > 0.05). At the phylum level, *Spirochaetota* was concentrated in the CD4^+^ and CD8^+^ low infiltration groups, *Proteobacteria* in the CD8^+^ high infiltration group, and *Actinobacteriota* in the FOXP3 low infiltration group, whereas no significant differences were found in the abundance of intratumoral microbiome in the PD-L1 subgroups (*P* > 0.05).

### 3.6 Relationship between intratumoral microbial load and clinical characteristics and TILs

To evaluate the potential value of intratumoral microbial load as a tumor biomarker, the microbial load of the OSCC group and the paracancerous control group was analyzed using the Wilcoxon rank sum test, and a significant difference was found between the bacterial load values of the OSCC group and those of the paracancerous control group (*P* < 0.05).The results of the χ^2^ test to analyzing the correlation between intratumoral microbial load and clinicopathological data did not reveal a significant correlation.

Finally, we analyzed the correlation between bacterial load and TILs markers (CD4^+^, CD8^+^, and FOXP3) expression by Spearman correlation analysis. It was found that the bacterial load was negatively correlated with CD4^+^, CD8^+^and FOXP3, but only the correlation coefficient of CD4^+^, -0.309, was statistically significant (*P*< 0.05).

## 4 Discussion

### 4.1 Intratumoral microbiome characteristics in OSCC

The oral cavity constitutes one of the most vital and complex microecosystems in the human body, harboring the second largest microbial community after the gut (14). Notably, the updated Hallmarks of Cancer framework identifies polymorphic microbiota as fundamental characteristics of tumor progression, capable of potentiating oncogenic hallmark traits. Dysbiotic microbial communities interact with the host genome to exert carcinogenic effects (15). In oral cancer, the intratumoral microbiome demonstrates particularly high biomass and exhibits strong associations with tumor microenvironment (TME) characteristics (16). Critically, microbial-immune cell interactions within the TME significantly modulate immune function, thereby influencing tumor initiation, progression, and therapeutic response (17, 18). In this study, we integrate cutting-edge microbiome profiling with comprehensive immunological analysis to systematically investigate the functional role of intratumoral microbiota in OSCC pathogenesis, providing a mechanistic foundation for advancing clinical diagnosis and treatment strategies.

In terms of α diversity, the OSCC group exhibited significantly reduced α diversity compared to normal controls (*P*<0.05). β diversity analysis further revealed substantial differences in microbial community structure between OSCC and normal groups (*P*<0.05), indicating profound microbiome alterations in OSCC tumors (19). However, neither α nor β diversity metrics showed significant differences between OSCC group and paracancerous group (*P*>0.05), consistent with previous reports (20). These findings suggest that the emergence of specific pathogenic microorganisms in OSCC disrupts ecological homeostasis, creating a dysbiotic tumor microbiome dominated by particular oncogenic species that may facilitate tumor progression.

At the phylum level, the OSCC group and the paracancerous control group had similar microbial composition ratios. From the perspective of change level, the abundance of Proteobacteria in the OSCC group increased compared with the paracancerous control group, while the abundance of Firmicutes and Bacteroidota decreased. The increased abundance of Proteobacteria has been reported to be a potential diagnostic feature of dysbiosis and disease risk (21). At the genus level, the dominant intratumoral microbiome in OSCC were mainly Fusobacterium, Prevotella, Streptococcus, Ralstonia, Neisseria, Pseudoalteromonas, Hemophilus, Bradyrhizobium, Porphyromonas, Leptotrichia, Acinetobacter, Veillonella, Campylobacter, Mycobacterium, and Actinomyces. Changes in microbiome may be related to the inflammatory and immune responses of the microenvironment. The tumorigenic effects of specific species of bacteria have been well established, especially Fusobacteriota, which has been described as having the function of “cancer bacillus” in a large number of reports on colorectal cancer (22). There are also reports of Streptococcus mitis, Prevotella melaninogenica and Porphyromonas gingivalis that have clear carcinogenic effects (20). Based on the above research results, OSCC intratumoral microbiome are different from normal oral microbiome, and dysbiosis is a risk factor for OSCC.

This study also found a significant correlation between microbial abundance and some clinical characteristics of OSCC patients (differentiation degree, T stage, lymph node metastasis, smoking history and drinking history). In previous studies on intratumoral microbiome, it was also found that the composition of intratumoral microbiome in OSCC at different stages and precancerous lesions was different (20), indicating that the composition of OSCC intratumoral microbiome changes with tumor progression. In addition, intratumoral microbiome can regulate the intrinsic properties of tumor cells and the external environment of cells, thereby promoting metastasis (23). Microbial similarities have been reported between primary tumors and metastatic lymph nodes in head and neck squamous cell carcinoma, which may be related to increased abundance of Proteobacteria (24), while selective elimination of metastasis-associated bacteria can inhibit tumor metastasis (25). OSCC risk factors such as smoking and drinking can change the bacterial acquisition and colonization of oral biofilms, thereby affecting the composition of the oral microbiome and the intratumoral microbiome. However, when OSCC was grouped by anatomical location (tongue, cheek, floor of mouth, palate and gums) for microbial abundance analysis, no changes were observed, indicating that the intratumoral microbiome of squamous cell carcinoma in the oral cavity is basically consistent and closely related to the oral microbiome. Studies have found that the intratumoral microbiome of HNSCC varies depending on the anatomical location (hypopharynx, oropharynx, nasopharynx, larynx, lip, tongue, tonsils, cricoid cartilage, and oral cavity, etc.) (26), different anatomical locations may lead to different microenvironments (27, 28). Although this study did not find a significant correlation between intratumoral bacterial load and clinical characteristics, some trends are still worth noting, such as the negative correlation between bacterial load and breast cancer stage in previous reports (29). The above correlation analysis between intratumoral microbiome and tumor clinical characteristics shows that the relationship between the microbiome and OSCC is still complex, and the mechanism of action of some microbiome has not been fully explained (30), and there is a lack of standardized microbial statistical methods in oral microbiome research (31).However, based on the above results, we can also observe that intratumoral microbiome interact with tumor progression to a certain extent.

Despite numerous recent studies investigating the association between HNSCC and intratumoral microbiome (32), the reported findings remain inconsistent - even for identical bacterial species. This discrepancy may be explained by the interdependent heterogeneity between the tumor microenvironment (TME) and the intratumoral microbiome. Significant variations in intratumoral microbiome composition can occur across different subtypes or stages of the same malignancy, underscoring how crucial microbiome-TME interactions are for tumor progression (33).Therefore, comprehensive studies of the intratumoral microbiome must incorporate multifactorial TME analyses. Furthermore, competitive interactions among microbial communities within tumors may lead to dynamic fluctuations in composition, as microbial abundances constantly shift through interspecies competition. For example, HPV-associated OSCC demonstrates markedly different intratumoral microbiome profiles compared to HPV-negative tumors, highlighting the impact of viral-microbiome interactions (34). Additionally, while substantial diversity exists at the bacterial genus level, current sequencing technologies cannot reliably resolve microbial classification to the species level (35). To date, beyond HPV-associated OSCC, no clinically validated intratumoral microbiome-derived biomarkers have been established for reliable prognosis prediction or treatment response evaluation in OSCC (36). Consequently, deeper investigation into intratumoral microbiome heterogeneity is essential to determine its influence on tumor progression and patient outcomes.

### 4.2 Intratumoral microbiome and the TME of OSCC

The most prominent immunological aberration in the HNSCC tumor microenvironment (TME) manifests as an altered ratio between effector T cells and regulatory T cells (Tregs), characterized by significantly elevated expression of the Treg transcription factor FOXP3 coupled with reduced CD4^+^ and CD8^+^ T cell infiltration (37). The immunomodulatory effects of the intratumoral microbiome further amplify the complexity of immune landscapes within the TME (38).

Our OSCC study demonstrates significant associations between specific microbiome profiles and CD4^+^/CD8^+^ T cell/FOXP3 Treg infiltration patterns. Moreover, the bacterial load demonstrated inverse correlations with CD4^+^, CD8^+^ T cell, and FOXP3^+^ Treg infiltration, among which the negative association with CD4+ T cells reached statistical significance. Although bacterial load was not significantly correlated with CD8^+^ T cell and FOXP3, which may potentially be attributed to limited sample size or random sampling variation, the trend is still worth noting. For example, previous studies on nasopharyngeal carcinoma have demonstrated that increased intratumoral microbial burden is associated with reduced T lymphocyte infiltration (39). A potential mechanistic explanation involves chronic antigen exposure from the intratumoral microbiome and its immunomodulatory metabolites fostering an immunosuppressive microenvironment that promotes T cell exhaustion (40) Studies have shown that microbial-associated molecular patterns can activate the NF-κB signaling pathway through Toll-like receptors, forming long-term chronic inflammation in the tumor microenvironment and ultimately promoting the formation of an immunosuppressive microenvironment (33).

Although our study did not detect significant correlations between PD-L1 expression and intratumoral microbial metrics—potentially due to limited sample size or stochastic variation—existing literature strongly implicates the microbiome in modulating immunotherapy responses (41). Specific groups of intratumoral microbes may be associated with immune checkpoint inhibitor (ICI) immunotherapy (42). For example, *γ-Proteobacteria* in non-small cell lung cancer tumors appear to downregulate PD-L1 and impair ICI efficacy (43), while preclinical models of HPV-associated oropharyngeal cancer identify oral microbiome signatures as predictive biomarkers for ICI outcomes (44). Mechanistically, intratumoral microbes can enhance antitumor immunity through cross-reactive antigen presentation (45, 46), CD8+ T cell recruitment (e.g., *Fusobacterium nucleatum* and *Bifidobacterium* spp (47–49)). Additionally, accumulating evidence indicates that intratumoral microbiota-derived metabolites actively participate in immune regulation (50). Specific bacterial metabolites can selectively accumulate near tumor cells and functionally remodel the TME (51), thereby modulating responses to immunotherapy. Also, research has demonstrated microbial regulation of the immune checkpoint protein CTLA-4 (52), where intratumoral bacteria promote CTLA-4 upregulation within immunosuppressive microenvironments, thereby impairing T cell infiltration into tumors (53). Beyond ICIs, tumor-associated microbial metabolites such as inosine have been shown to potentiate the functionality of chimeric antigen receptor (CAR) T cells, suggesting their potential to enhance the efficacy of immunotherapeutic approaches (54).

A growing body of evidence supports the modulation of intratumoral microbiota as a promising adjunctive strategy in cancer therapy, where specific microbial communities promote an immunosuppressive TME and contribute to treatment resistance (55). Therapeutic approaches targeting bacterial-derived peptides (56) or reshaping microbial composition through probiotics may enhance immunotherapy efficacy (57). For example, targeted bacterial ablation in pancreatic cancer promotes CD4^+^ T cell differentiation and CD8^+^ T cell activation while upregulating PD-1 to improve ICI response (58). Elimination of *Fusobacterium nucleatum* in breast cancer enhances ICI effectiveness (59). Combining *Megasphaera* with ICIs yields superior tumor suppression in pancreatic ductal adenocarcinoma (60), and *Bifidobacterium* supplementation with ICIs induces near-complete tumor regression in melanoma (61). Moreover, *Lactobacillus acidophilus* exhibits direct antiproliferative effects against OSCC cells (62). Currently, there are also a variety of bacteria-mediated cancer immunotherapies entering the clinical development stage (63).

In summary, as an indispensable component of the TME, the intratumoral microbiome promotes pro-tumor inflammation and induces T cell dysfunction, thereby attenuating antitumor immune responses and compromising immunotherapy efficacy. These findings position specific intratumoral microbiota as promising therapeutic targets, with modulation of the immune-oncology-microbiome axis offering new dimensions to current treatment strategies (6, 64). Although PD-1/PD-L1 inhibitors are guideline-recommended for advanced HNSCC, and PD-L1 expression (CPS/TPS) remains the sole validated predictive biomarker, only ∼20% of patients achieve durable responses (65–67). This highlights the imperfect predictive value of PD-L1, as even CPS-high patients may show primary resistance (65, 66, 68, 69). Overreliance on CPS may deny therapy to potential responders while risking immune-related adverse events or hyperprogression in others (70). Future approaches should integrate PD-L1 status with multifactorial assessments, potentially incorporating intratumoral microbiome signatures to better identify responders. Targeted microbiome modulation may further improve outcomes, potentially benefiting broader patient populations.

## Data Availability

The data presented in the study are deposited in the NCBI Sequence Read Archive (SRA) repository, accession number PRJNA 1328350.
